# Exploring Evidence of Non-coding RNA Translation With Trips-Viz and GWIPS-Viz Browsers

**DOI:** 10.3389/fcell.2021.703374

**Published:** 2021-08-12

**Authors:** Oza Zaheed, Stephen J. Kiniry, Pavel V. Baranov, Kellie Dean

**Affiliations:** ^1^School of Biochemistry and Cell Biology, University College Cork, Cork, Ireland; ^2^Shemyakin-Ovchinnikov Institute of Bioorganic Chemistry, RAS, Moscow, Russia

**Keywords:** translation, ribosome profiling, Ribo-seq, small open reading frames (smORFs), non-coding RNAs, LncRNA - long noncoding RNA, microprotein, RNA-Seq

## Abstract

Detection of translation in so-called non-coding RNA provides an opportunity for identification of novel bioactive peptides and microproteins. The main methods used for these purposes are ribosome profiling and mass spectrometry. A number of publicly available datasets already exist for a substantial number of different cell types grown under various conditions, and public data mining is an attractive strategy for identification of translation in non-coding RNAs. Since the analysis of publicly available data requires intensive data processing, several data resources have been created recently for exploring processed publicly available data, such as OpenProt, GWIPS-viz, and Trips-Viz. In this work we provide a detailed demonstration of how to use the latter two tools for exploring experimental evidence for translation of RNAs hitherto classified as non-coding. For this purpose, we use a set of transcripts with substantially different patterns of ribosome footprint distributions. We discuss how certain features of these patterns can be used as evidence for or against genuine translation. During our analysis we concluded that the *MTLN* mRNA, previously misannotated as lncRNA *LINC00116*, likely encodes only a short proteoform expressed from shorter RNA transcript variants.

## Introduction

Ribosome profiling, or footprinting (a.k.a. Ribo-seq), has allowed for a detailed assessment of whole cellular transcriptomes (Ingolia et al., 2009). The Ribo-seq technique enables this by generating a snapshot of active ribosome locations at a given moment by only sequencing the parts of RNA molecules protected by the ribosome during translation, which are termed ribosome protected fragments (RPFs) or ribosome footprints (Ingolia, 2014). These data are used for inferring parameters of translation, including translation rates of individual mRNAs, differential translation, ribosome pause detection and identification of translated open reading frames (ORFs), among others (Ingolia, 2014; Brar and Weissman, 2015; Andreev et al., 2017). A plethora of computational approaches and software tools have been developed for the analysis of ribosome profiling data (Kiniry et al., 2020). Among many findings made with the use of ribosome profiling were observations of translation of some of the RNA molecules that were previously classified as non-coding RNA (ncRNA).

The term “non-coding RNA” had classically referred to a very large and diverse group of RNA molecules that number in the thousands (Cheng et al., 2005; Birney et al., 2007; Washietl et al., 2007; van Bakel et al., 2010; Shahrouki and Larsson, 2012). Within non-coding RNA, RNAs longer than 200 nucleotides were classified as long non-coding RNA (lncRNA), while shorter transcripts were referred to as small RNAs. These small RNAs were sub-divided into transfer RNAs (tRNAs), micro RNAs (miRs), short-interfering RNAs (siRNAs), piwi interacting RNAs (piRNAs) etc. (Storz, 2002; Großhans and Filipowicz, 2008; Fang and Fullwood, 2016).

The evidence that lncRNAs can be translated was initially provided by Ingolia et al. (2011). Later, by analyzing available data, Chew et al., demonstrated that the high ribosomal occupancy in many lncRNAs resembles that in 5′ leaders of protein coding mRNAs (Chew et al., 2013). The 5′ leader sequences often contain translated short open reading frames, providing an argument in support of translation within lncRNAs. A counter argument was made by Guttman et al., who used ribosome footprint density at stop codons as a signature of genuine translation and developed ribosome release score (RRS) to measure it (Guttman et al., 2013). High RRSs are observed for long protein coding ORFs, but not for short ORFs in 5′ leaders and lncRNAs. This argument, however, is flawed, as it only shows that re-initiation and leaky scanning are infrequent downstream of long protein coding ORFs. Indeed, translation of downstream ORFs is observed only in rare cases downstream of relatively short ORFs lacking ATG codons within the entire coding sequence (Benitez-Cantos et al., 2020) or during equally infrequent stop codon readthrough (Loughran et al., 2014). However, when ORFs are short and their translation is inefficient, re-initiation (Munzarová et al., 2011) and leaky scanning (Michel et al., 2014a) are possible, so that the 5′ leaders and lncRNAs could have multiple, often overlapping ORFs that are translated. Subsequently, Ingolia et al. (2014) developed an approach for discriminating genuine translation from aberrant RNA protection by the ribosome or other large ribonucleoprotein complexes with the analysis of the distribution of ribosome footprint lengths, called the fragment length organization similarity score, FLOSS. FLOSS scores appear to be similar for protein coding ORFs, 5′ leaders and lncRNAs, but were distinct for the protected fragments derived from RNAs with known non-coding functions (Ingolia et al., 2014). While there is an overwhelming body of evidence that many lncRNAs have translated ORFs, it is unlikely that many of them code for stable protein products because the lack of long ORFs and of nucleotide substitution patterns typical for protein coding evolution. Although the functional significance of the translated ORFs remains largely unclear, emerging data suggest certain possibilities, such as ribosome assisted RNA processing (Sun et al., 2020).

Several mRNAs coding for small proteins were initially misclassified as lncRNAs, and some of them were “upgraded” to the status of mRNAs after their products have been identified and characterized. An example is *LINC00116* that was found to code for a 56-amino acid functional microprotein found in mitochondria (Catherman et al., 2013). Later, it was independently rediscovered and characterized by several groups, named mitoregulin and assigned the protein coding gene symbol *MTLN* (Stein et al., 2018; Chugunova et al., 2019; Lin et al., 2019). Mitoregulin has been shown to enhance mitochondrial respiratory activity (Chugunova et al., 2019) and play a regulatory role in adipocyte metabolism (Friesen et al., 2020).

There are many other examples of microprotein encoding mRNAs misclassified as non-coding; a 46-amino acid microprotein, myoregulin encoded by *LINC00948* (Anderson et al., 2015); the 7-kilodalton microprotein, non-annotated P-body dissociating polypeptide (NoBody) encoded by *LINC01420* (D’Lima et al., 2017); the microprotein, CIP2A-BP encoded by *LINC00665* that inhibits triple negative breast cancer progression (Guo et al., 2020), and the small endogenous peptide, SMIM30, which promotes hepatocellular cancer tumorigenesis, encoded by *LINC00998* (Pang et al., 2020) to name a few. Nonetheless, it is likely that more await discovery, and therefore analysis of lncRNA translation and protein coding potential is an active area of research.

A number of tools have been developed for automatic detection of translated ORFs using Ribo-seq data (Crappé et al., 2015; Fields et al., 2015; Ji et al., 2015; Raj et al., 2016; Reuter et al., 2016; Erhard et al., 2018; Xiao et al., 2018; Brunet et al., 2019), their predictions vary and in the absence of a gold standard, their accuracies are difficult to estimate (Baranov and Michel, 2016). RNA protection from nuclease digestion could also occur from large RNA-protein complexes other than the ribosome. In fact, a tool Rfoot has been developed specifically for identification of such RNase protection due to RNA-binding proteins (Ji, 2018). It has been discussed that ribosomal footprints can be differentiated from non-ribosomal activity *via* differences in footprint length and lack of triplet periodicity (Ji et al., 2016; Ingolia et al., 2019). Therefore, for accurate and reliable detection of genuinely translated ORFs and protein-coding potential, it is often necessary to carefully examine available data manually. Here, we demonstrate how publicly available ribosome profiling data can be explored using ribosome profiling data resources from RiboSeq. Org portal, Trips-Viz (TRanscriptome-wide Information on Protein Synthesis-Visualized) and GWIPS-viz (Genome Wide Information on Protein Synthesis-visualized).

Trips-Viz is a graphical user interface (GUI) on-line platform that allows for interactive analysis and visualization of Ribo-seq and shotgun RNA sequencing (RNA-seq) data aligned to transcriptomes (Kiniry et al., 2019, 2021). To date Trips-Viz contains 2064 Ribo-seq files and 752 RNA-seq files from 114 studies across nine organisms. In the section “Setup and Configurations,” we describe in detail how to use the relevant functionalities of Trips-Viz. In the section “Data exploration in the context of individual RNA sequences,” we examine a selection of transcripts that illustrate different patterns of ribosomal footprints aligned to them and evaluate these patterns for genuine translation, see Table 1. The GWIPS-viz browser provides visualization of unambiguously mapped footprints to reference genomes (Michel et al., 2014b) and its use is necessary in order to evaluate how well transcript annotations and gene structures are supported by available data. In addition, we use the codon alignment viewer (CodAlignView) that is helpful for visualization of codon substitution that can reflect evolutionary selection acting on protein coding sequences (Jungreis et al., 2021).

**TABLE 1:** List of RNAs examined with the corresponding accession number and transcript coordinates of the ORFs in order of appearance.

**Gene**	**Accession number**	**Start**	**Stop**
*MTLN*	ENST00000414416	1,401	1815
*RPPH1*	ENST00000554988	257	356
*SNHG8*	ENST00000602414	112	270
*SNHG8*	ENST00000602483	93	201
*SNORA24*	ENST00000384096	51	111
*ZFAS1*	ENST00000428008	122	197
*ZFAS1*	ENST00000428008	15	39
*SNORD12C*	ENST00000386307	–	–
*XIST*	ENST00000429829	138	167
*XIST*	ENST00000429829	765	879

## Setup and Configurations

Trips-Viz^1^ provides data aligned to the transcriptomes of several organisms and a rich repertoire of functional visualizations for the analysis of ribosome profiling data. Here we focus on *Homo sapiens* and the function “Single transcript plot” to manually examine transcripts of interest. For further explanation on the other analyses available within Trips-Viz, please refer to detailed instructions and videos available within the Trips-Viz platform (Kiniry et al., 2019, 2021).

Using prior knowledge, we selected the translated ORF on *MTLN* mRNA (formerly *LINC00116*) to serve as an example for genuine translation. As an example of a ncRNA whose ORF translation is unlikely we chose *RPPH1* that encodes for the RNA component of RNase P. We further explored ribosome footprints aligned to *SNHG8*, *ZFAS1*, and *XIST*. The translation of all three lncRNAs has been reported previously (Ji et al., 2015; Calviello et al., 2016; Martinez et al., 2020), the translation of *SNHG8* and *ZFAS1* was also reported in additional studies (van Heesch et al., 2019; Gaertner et al., 2020) and the translation of *ZFAS1* was also reported by Chen et al. (2020).

While the default options for “Single transcript plot” are usually adequate for initial analysis, there are several parameters that could affect the analysis and their meaning needs to be explained. “Min triplet periodicity score” is a threshold used to filter the data based on the strength of triplet periodicity signal. Triplet periodicity can be used for identification of the reading frame of translation (Michel et al., 2012). Triplet periodicity, as well as other parameters of ribosome profiling data, vary considerably across different studies (O’Connor et al., 2016). Therefore, not all data offer the same power to accurately identify the translated reading frame. To improve the quality of this parameter we used a triplet periodicity score cutoff of 0.5, meaning any read lengths with a score less than this would not be displayed. In addition to improving detection of the footprints’ frame of origin, good triplet periodicity is also an indirect signature of good data quality. Although reducing the number of reads analyzed does reduce the coverage and potentially exclude the detection of certain lowly translated ORFs, a reasonably large number of Ribo-seq datasets pass the 0.5 threshold, see Table 2.

**TABLE 2:** List of studies used for the ‘single transcript plot’ analysis with corresponding triplet periodicity scores, cell line/tissue and number of samples for each study (Guo et al., 2014; Wolfe et al., 2014; Crappé et al., 2015; Werner et al., 2015; Calviello et al., 2016; Goodarzi et al., 2016; Iwasaki et al., 2016; Ji et al., 2016; Park et al., 2016; Xu et al., 2016; Fija-Lkowska et al., 2017; Zhang et al., 2017; Gameiro and Struhl, 2018).

**Study**	**Triplet periodicity**	**Cell line/tissue**	**Number of samples**
Werner 15	0.55	H1	23 RNA-seq, 23 Ribo-seq
Gameiro 18	0.57	MCF10A	25 RNA-seq, 25 Ribo-seq
Park 16	0.71	HeLa	9 RNA-seq, 7 Ribo-seq
Guo 14	0.74	U2OS	6 RNA-seq, 3 Ribo-seq
Zhang 17	0.61	HEK293	3 Ribo-seq
Calviello 16	0.79	HEK293	1 Ribo-seq
Fijalkawska 17	0.66	HCT116	2 RNA-seq, 4 Ribo-seq
Xu 16	0.71	Human wild type fibroblasts, *ESCO2*-corrected Robert’s Syndrome fibroblasts, *ESCO2-*mutant Robert’s Syndrome fibroblasts	22 RNA-seq, 20 Ribo-seq
Ji 16	0.58	MCF10A-ER-Src, BJ	10 RNA-seq, 26 Ribo-seq
Wolfe 14	0.68	KOPT-K1 T-ALL	18 RNA-seq, 4 Ribo-seq
Crappe 15	0.68	HCT116	2 Ribo-seq
Iwasaki 16	0.56	HEK293 Flp-In T-Rex	5 RNA-seq, 10 Ribo-seq
Goodarzi 16	0.58	MDA-parental	23 RNA-seq, 24 Ribo-seq

Another important parameter is the use of ambiguously mapped reads. Ribosome footprints are short and therefore often cannot be unambiguously aligned. Enabling such multimapping creates an uncertainty regarding the true origin of the footprint. However, disabling multimapping results in a reduction of footprint density in the areas that share similarity with other sequences from the same genome. A number of approaches to mitigate this issue has been developed, see Kiniry, Michel and Baranov for a review (Kiniry et al., 2020). Trips-Viz, however, can either enable or disable ambiguous reads mapping. Here we disable multimapping by default to maximize the specificity, but sometimes explore ribosome profiling density plots under both modes, as this may help in interpretation of data for genes occurring in multiple copies and for closely related paralogs. In addition, when available, the corresponding RNA-seq studies were also enabled. Distribution of RNA-seq reads can be used to assess whether the annotation of a transcript is supported by the data, as well as to assess the mappability of corresponding regions. Changes in RNA-seq coverage could indicate regions that are difficult to sequence or to align, although RNA-seq data can exhibit its own RNA-seq specific biases, such as an increase of density toward the 3′end due to preferential capture of polyadenylated RNA fragments when poly-dT is used for mRNA capture (Weinberg et al., 2016). We visualized exon locations by enabling “Exon Junctions” on the generated plot legends tab which makes it easier to track in conjunction with genomic alignments. Finally in some individual cases, we also used mass spectrometry data available in Trips-Viz.

For visualization of genomic alignments, assessment of gene structures and selection of most appropriate transcript isoforms, we used the GWIPS-viz browser. Unlike Trips-Viz, GWIPS-viz provides ribosome profiling data aligned to the reference genome sequences, instead of transcriptome sequences. GWIPS-viz is based on the UCSC genome browser (Navarro Gonzalez et al., 2021) and is easy to use for anyone familiar with the latter. In addition to ribosome profiling data tracks, GWIPS-viz provides a number of auxiliary tracks that are helpful in the interpretation of ribosome profiling data, such as annotation tracks. Here we used the following tracks: “Basic Annotation Set from Gencode Version 25”; “mRNA-seq coverage from all studies,” which is a global aggregate for the number of RNA-seq reads aligned to each coordinate; “Ribosome profiles from all studies,” which is the visualization of inferred coordinates of ribosome A-sites (elongating ribosomes); “Initiating ribosome profiles from all studies” is the track for P-sites of ribosomes captured with translation inhibitors that preferentially arrest initiating ribosomes (Ingolia et al., 2011; Lee et al., 2012). Finally, we also enabled “Basewise Conservation by PhyloP100way” for assessment of nucleotide sequence conservation (Pollard et al., 2010). The default color scheme: elongating ribosome profiles are shown in red; initiating ribosome profiles are in blue; while, RNA-seq data are in green (Supplementary Figure 1A). It is important to note that while Trips-Viz alignments are strand-specific since transcripts are single stranded, GWIPS-viz alignments are not strand specific. Strand-specificity is provided only for bacterial genomes where a large proportion of genes overlap and the data interpretation would be difficult otherwise. Strand-specificity is provided only for bacterial genomes where a large proportion of genes overlap and the data interpretation would be difficult otherwise. Translation of overlapping antisense lncRNAs has been reported in mammals (Ruiz-Orera and Albà, 2019); hence, to properly analyze the corresponding loci, it is important to explore the corresponding RNAs in Trips-Viz.

In addition to ribosome profiling data resources, we took advantage of CodAlignView^2^, which differentially colors synonymous and non-synonymous codon substitutions, while also differentially coloring the latter depending on whether they lead to similar or radical changes according to BLOSUM62 (Jungreis et al., 2021). Such visualizations enable manual exploration of evolutionary selection acting on potential protein coding sequences, as synonymous and conservative non-synonymous substitutions are more frequent in protein coding sequences than radical, non-synonymous substitutions (M. F. Lin et al., 2011). The tool also differentially highlights stop codons and ATG codons, and visualizes other features such as predicted splice sites (Supplementary Figure 1B).

## Data Exploration in the Context of Individual RNA Sequences

### *MTLN* mRNA as an Example of Genuine Protein-Producing Translation

As mentioned earlier, *MTLN* was previously misannotated as lncRNA *LINC00116*. Since its productive translation has been extensively characterized, we used it as a “gold standard” example.

**FIGURE 1:**
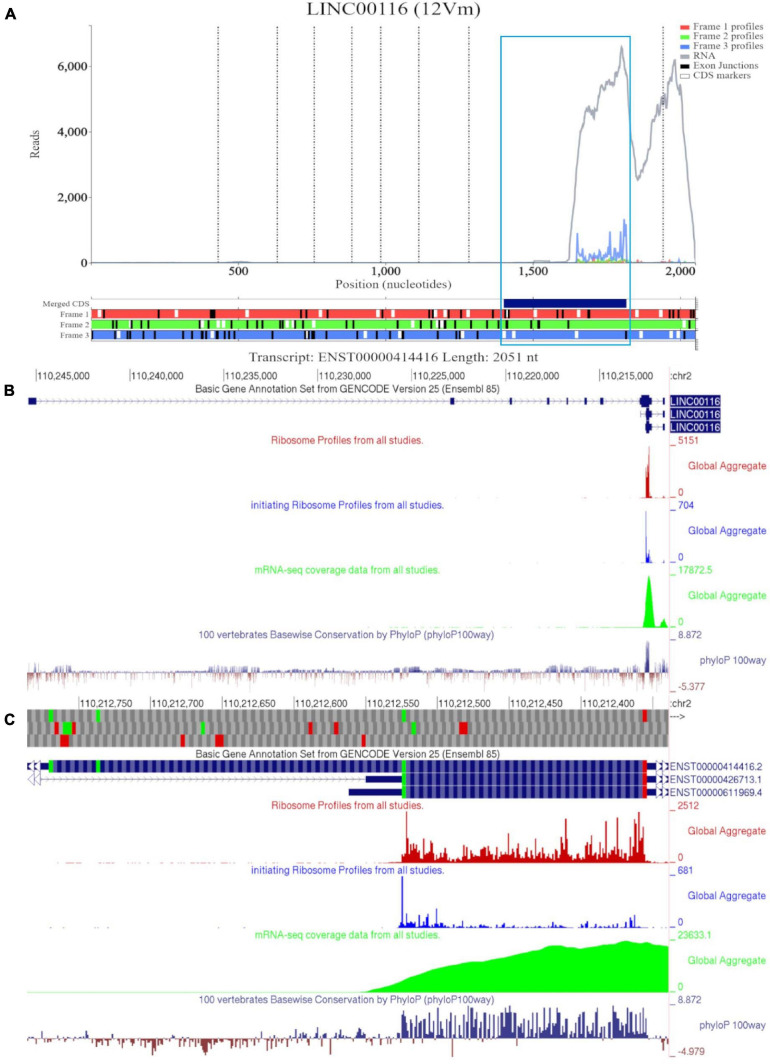
Visualization of *MTLN/LINC00116* in Trips-Viz and GWIPS-Viz. **(A)** Whole transcript view of *MTLN* in Trips-Viz with data indicating the protein product is shorter than the annotated coding sequence. **(B)** Visualization of *MTLN/LINC00116* in GWIPS-Viz with the ‘Basic Gene Annotation Set from Gencode Version 25,’ ‘Ribosome Profiles from all studies,’ ‘initiating Ribosome Profiles from all studies,’ ‘mRNA-seq coverage data from all studies’ and ‘100 vertebrates Basewise Conservation by PhyloP100way’ tracks enabled. **(C)** Coding sequence on *MTLN* in GWIPS-viz with the tracks described in Setup and Configurations enabled.

Figure 1A shows RNA-seq and Ribo-seq data aligned to the sequence of longest *MTLN* mRNA isoform (ENST00000414416). However, there appears to be no RNA-seq coverage for most of the annotated sequence up to ∼1500 nucleotides (nt) downstream of the annotated transcript 5′ end. For reference, we have also included a Trips-Viz visualization with ambiguously mapped reads enabled (Supplementary Figure 2A). When ambiguous mapping is allowed, only an isolated peak of RNA-seq emerges in the region of the second exon, ∼500 nt. The discontinuous RNA-seq coverage strongly suggest that this peak is an artifact of ambiguous mapping. Thus, the data suggest that a much shorter transcript is transcribed in all cells that were used for producing these data (Table 2). Consistent with that, ribosome profiling data appears only downstream of the fifth ATG in the annotated CDS (third in CDS frame). Indeed, if the entire annotated transcript were to be translated, how would preinitiation ribosome complexes reach the annotated coding sequence (CDS), bypassing ∼25 ATGs upstream? Existence of a shorter transcript explains this conundrum as the fifth ATG in the annotated CDS appears to be the first ATG in the truncated transcript supported by both the RNA-seq and Ribo-seq data, furthermore initiation at this ATG would be expected under the classic scanning model of translation initiation. Triplet periodicity strongly supports translation of the annotated CDS frame (frame three) indicating the genuine “translational” nature of Ribo-seq reads. Trips-Viz also contains proteomics data on the peptide masses that can be matched in mass-spectrometry datasets using MSFragger (Kong et al., 2017) and Philosopher (da Veiga Leprevost et al., 2020). Supplementary Figure 2B shows a screenshot of available data. Interestingly, while the most abundant peptides match *MTLN* CDS (in blue), there are also peptides whose masses matches products of conceptual translation of other reading frames (green and red), they may represent false positives.

**FIGURE 2:**
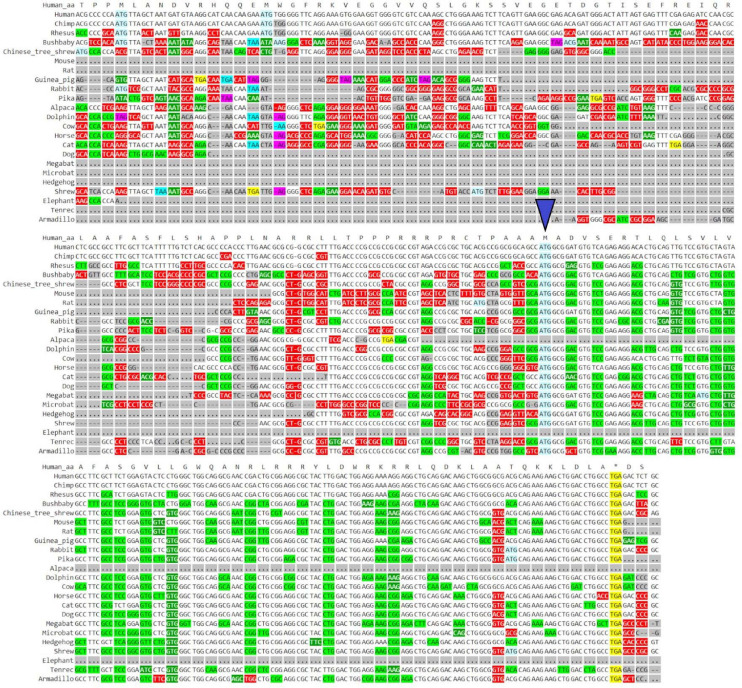
Visualization of *MTLN/LINC00116* in CodAlignView. Coding sequence of *MTLN* visualized in CodAlignView with a blue triangle pointing toward the third ATG start site which correlates with the region where a high ratio of synonymous codon substitutions begins.

GWIPS-viz can be used to further explore whether the annotated transcript is supported by available RNA-seq and Ribo-seq data. For example, it is possible that some of the annotated introns are retained in mature RNA transcripts and would not be represented in Gencode and subsequently in Trips-Viz. Since the data are aligned to the genomes in GWIPS-viz, such problems with transcript annotations can be spotted. GWIPS-viz also provides an easy way to examine which RNA isoform is best supported by the data when multiple isoforms are present. The analysis of the *MTLN* locus on GWIPS-viz (Figure 1B) did not reveal the presence of RNA-seq or Ribo-seq reads in addition to what is seen in Trips-Viz. Further, it can be seen that in addition to the long isoform, there are two additional short isoforms (ENST00000426713 and ENST00000611969), with annotated CDS starts from the same start codon that we proposed on the analysis of data in Trips-Viz. Figure 1C shows an enlarged view of this area. A high peak of footprints obtained by enriching ribosomes at the initiating sites can be seen to match the same ATG. The same region also displays high nucleotide conservation in the PhyloP track with a pattern of triplet periodicity typical to protein coding regions due to higher frequency of substitutions in the third subcodon position relative to the first and second subcodon positions. The substitution patterns can be explored more reliably with CodAlignView (Figure 2), where a white color indicates absolute nucleotide conservation; while predominance of green (synonymous or conservative substitutions) is reflective of protein coding evolution. Yet again, the “green” region coincides with the region of high Ribo-seq density observed in the CDSs of shorter RNA isoforms. For reference, the alignment set used in CodAlignView was the 24-mammal subset of the 100-way vertebrate alignment using the hg38 human genome assembly (Rosenbloom et al., 2015). Of note, it was the shorter proteoform that was detected and characterized in previous studies (Catherman et al., 2013; Stein et al., 2018; Chugunova et al., 2019; Lin et al., 2019).

In summary, the translation of a short proteoform from the short RNA isoforms of *MTLN* gene is supported by all types of data explored here. This provides a good reference point for the case of genuine translation resulting in production of a stable protein.

### RNase P RNA as an Example of Untranslated RNA

RNase P is a large nucleoprotein complex responsible for processing many RNA molecules (Evans et al., 2006). The RNA component of RNase P is transcribed by polymerase III and therefore is not capped (Schramm and Hernandez, 2002). Thus, it is extremely unlikely to be translated, yet fragments of RNase P RNA could contaminate Ribo-Seq data due to protection within the complex and co-isolation with ribosomes. Therefore, we chose the RNA component of RNase P as an example of an untranslated non-coding RNA. In humans it is encoded by the *RPPH1* gene.

**FIGURE 3:**
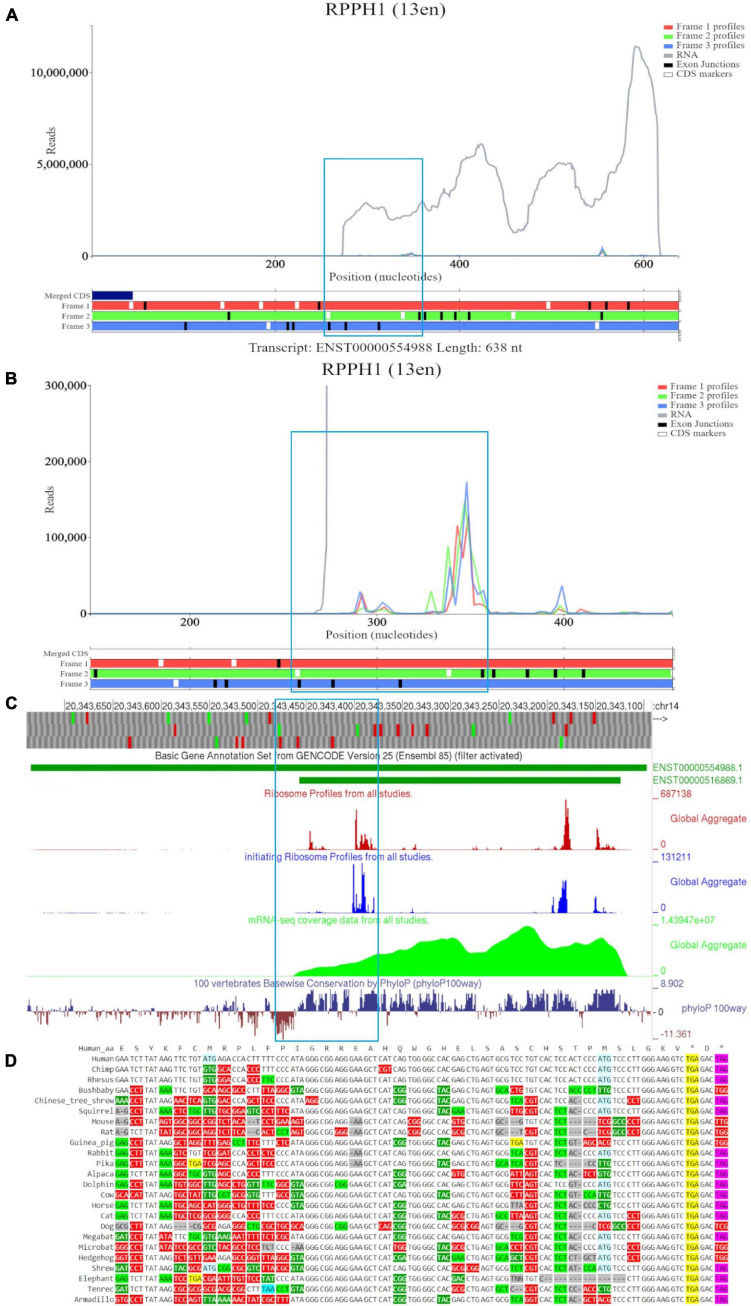
Visualization of *RPPH1* in Trips-Viz, GWIPS-Viz and CodAlignView. **(A)** Whole transcript view of *RPPH1* in Trips-Viz with the ORF outlined in blue. **(B)** Enlarged view of the ORF on *RPPH1* in Trips-Viz **(C)** Visualization of the *RPPH1* in GWIPS-viz with the tracks described in Setup and Configurations enabled. **(D)** CodAlignView of the ORF on *RPPH1* showing predominantly absolute codon conservation.

Figure 3A shows a Trips-Viz screenshot displaying the data aligned to the long RNA isoform *RPPH1* (ENST00000554988). Like in the previous case, only part of the annotated transcript is supported by RNA-seq data as visualized in the GWIPS-viz browser (Figure 3C), indicating the presence of the shorter transcript isoform (ENST00000516869). There are several isolated peaks of ribosome footprint density across the transcript that do not correspond to a single ORF. One of the longest ORFs, with the largest number of footprints aligned to it, is in the second (green) reading frame and is depicted within a blue rectangle on Figure 3A. It can be explored at higher magnification in Figure 3B. The mapped reads do not show any triplet periodicity, indicating there is no preferential support for a specific reading frame. The PhyloP track in GWIPS-viz (Figure 3C) indicates high, nucleotide conservation expected for the sequence of this important housekeeping RNA molecule. However, it does not exhibit a pattern characteristic for protein coding evolution (prevalence of synonymous and positive non-synonymous codon substitutions over radical non-synonymous substitutions, see Figure 3D). Thus, *RPPH1* represents a genuine example of an untranslated non-coding RNA, with aligned Ribo-seq data that most likely has origins other than protection by translating ribosomes.

### Examples of Translation That Are Unlikely to Produce Proteins

For the exploration of translation of lncRNAs whose translational status is less clear, we chose *SNHG8, ZFAS1*, and *XIST*. Their translation has been previously reported by several independent ribosome profiling studies (Ji et al., 2015; Calviello et al., 2016; van Heesch et al., 2019; Chen et al., 2020; Gaertner et al., 2020; Martinez et al., 2020) using different methods for automatic detection of translated ORFs (Fields et al., 2015; Calviello et al., 2016; Ji, 2018).

For this analysis, we started with small nucleolar RNA host gene 8 (*SNHG8*), a lncRNA located on human chromosome 4q26. This lncRNA hosts the H/ACA-box small nucleolar RNA (snoRNA), *SNORA24*. Non-coding genes that host snoRNAs were found to have short, poorly conserved ORFs and were believed to serve little function outside of carrying snoRNAs in their introns (Tycowski et al., 1996; Smith and Steitz, 1998).

**FIGURE 4:**
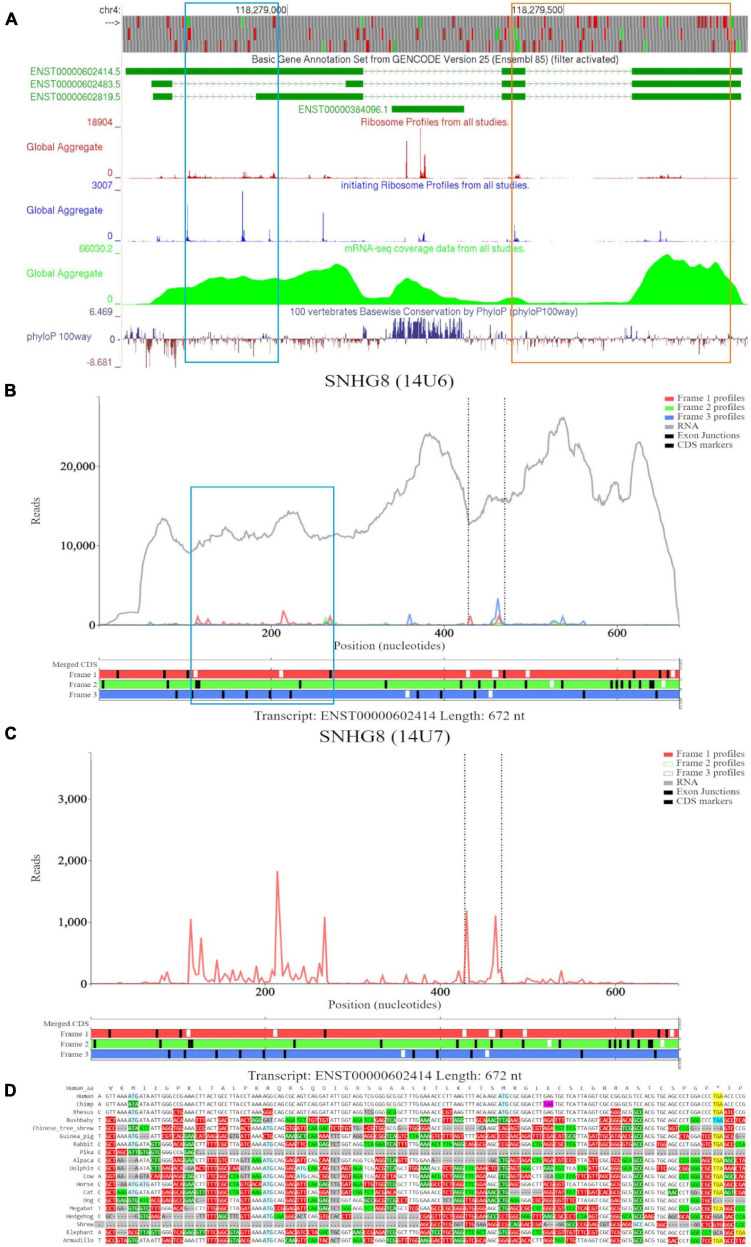
Visualization of *SNHG8* in Trips-Viz, GWIPS-Viz and CodAlignView. **(A)** Visualization of *SNHG8* in GWIPS-viz with the tracks described in Setup and Configurations enabled. Two translated ORFs are outlined in blue and orange, respectively. **(B)** Visualization of *SNHG8* isoform ENST00000602414 in Trips-viz. The ORF previously outlined in blue in GWIPS-viz is similarly outlined again. **(C)** Visualization of transcript ENST00000602414 without RNA-seq data enabled and only reading frame 1 enabled. **(D)** CodAlignView for the ORF previously outlined in blue showing a high ratio of radical non-synonymous codon substitutions.

Examination of *SNHG8* in GWIPS-viz reveals three isoforms ENST00000602414, ENST00000602483, and ENST00000602819 (Figure 4A). The first two ATGs match with high footprint peaks of initiating ribosomes and are outlined in blue. Nucleotide conservation at this locus is poor, and a signature of accelerated evolution is seen on the PhyloP track. RNA-seq data suggests that the long isoform ENST00000602414 is most likely transcribed. The eighth ATG (outlined in orange) also matches a high footprint peak of initiating ribosomes. Nucleotide conservation for this ORF is similarly poor as visualized on the PhyloP track. It should be noted that all three RNA isoforms contain this ORF. However, initiation at the eighth ATG is more likely under the classical scanning model on the shorter isoform ENST00000602483, as it is the second ATG from the 5′ end. We also note another footprint peak of initiating ribosomes that matches with the ATG (sixth ATG site) located on *SNORA24* (ENST00000384096); yet, elongating ribosome footprints would not fully encompass the ORF situated at this locus. The PhyloP tracks reveals high nucleotide conservation at *SNORA24* indicating its important functional role.

Based on the features seen on GWIPS-viz, we first examined transcript ENST00000602414 on Trips-Viz (Figure 4B). Footprints aligned at the first ATG show good triplet periodicity with the reads biased to reading frame one (red). This signal is better visualized in Figure 4C with removal of the footprints supporting other reading frames. The corresponding region is shown in CodAlignView in the reading frame matching the ORF (Figure 4D), the high density of radical codon substitutions is not supportive of protein coding evolution.

**FIGURE 5:**
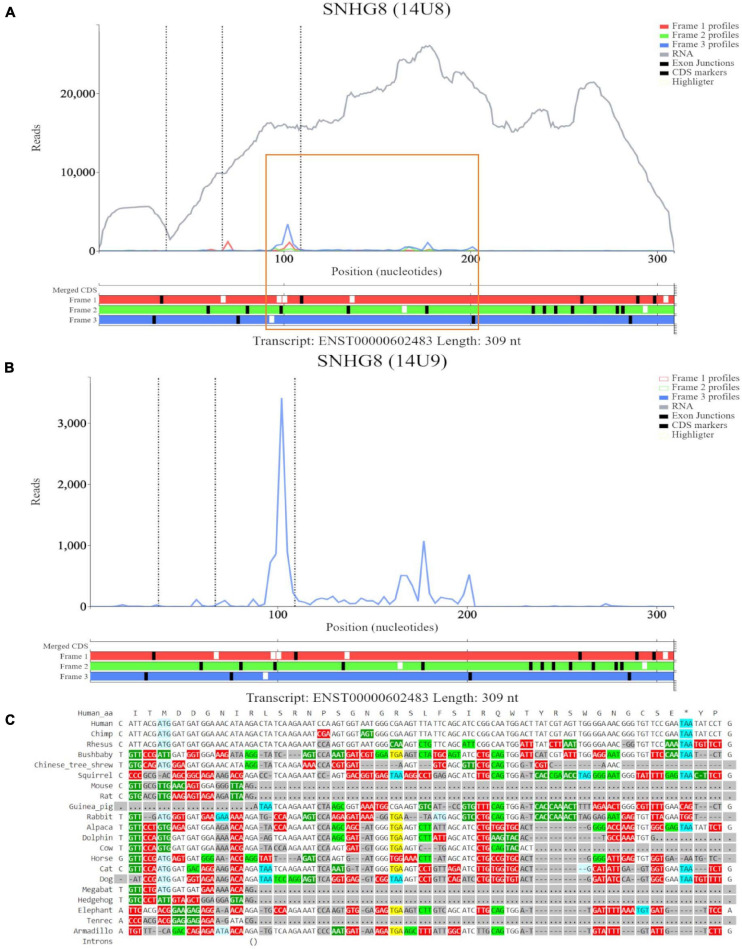
Visualization of *SNHG8* in Trips-Viz and CodAlignView. **(A)** Visualization of *SNHG8* isoform ENST00000602483 in Trips-viz. The ORF previously outlined in orange in GWIPS-viz is similarly outlined again. **(B)** Visualization of transcript ENST00000602483 without RNA-seq data enabled and only reading frame 3 enabled. **(C)** CodAlignView for the ORF previously outlined in orange showing a high ratio of radical non-synonymous codon substitutions.

For the ORF at the eighth ATG noted on GWIPS-viz, we examined transcript ENST00000602483 in Trips-Viz (Figure 5A). Ribosomal footprints appear aligned to the second ATG and support reading frame three (blue). This region is shown at close zoom on Figure 5B. However, codon substitution pattern (Figure 5C) is not supportive of translation.

For completion, we visualized *SNORA24* in Trips-Viz (Supplementary Figure 3A). Although there are footprints aligned to the ATG site that are biased to a single reading frame (blue), they do not encompass the length of the ORF. Small nucleolar RNAs function in ribosome biogenesis and therefore are likely to be isolated as parts of inactive ribosomal complexes. It is also possible that they are protected within other RNA-protein complexes (Ji et al., 2016).

The next RNA examined was zinc finger antisense 1 transcript (*ZFAS1*), a lncRNA located on human chromosome 20q13.13. It is positioned at the antisense strand of the 5′ end of the protein coding *ZNFX1* gene. *ZFAS1* also hosts three C/D-box snoRNAs namely *SNORD12C*, *SNORD12B*, and *SNORD12* in sequential introns (Askarian-Amiri et al., 2011).

**FIGURE 6:**
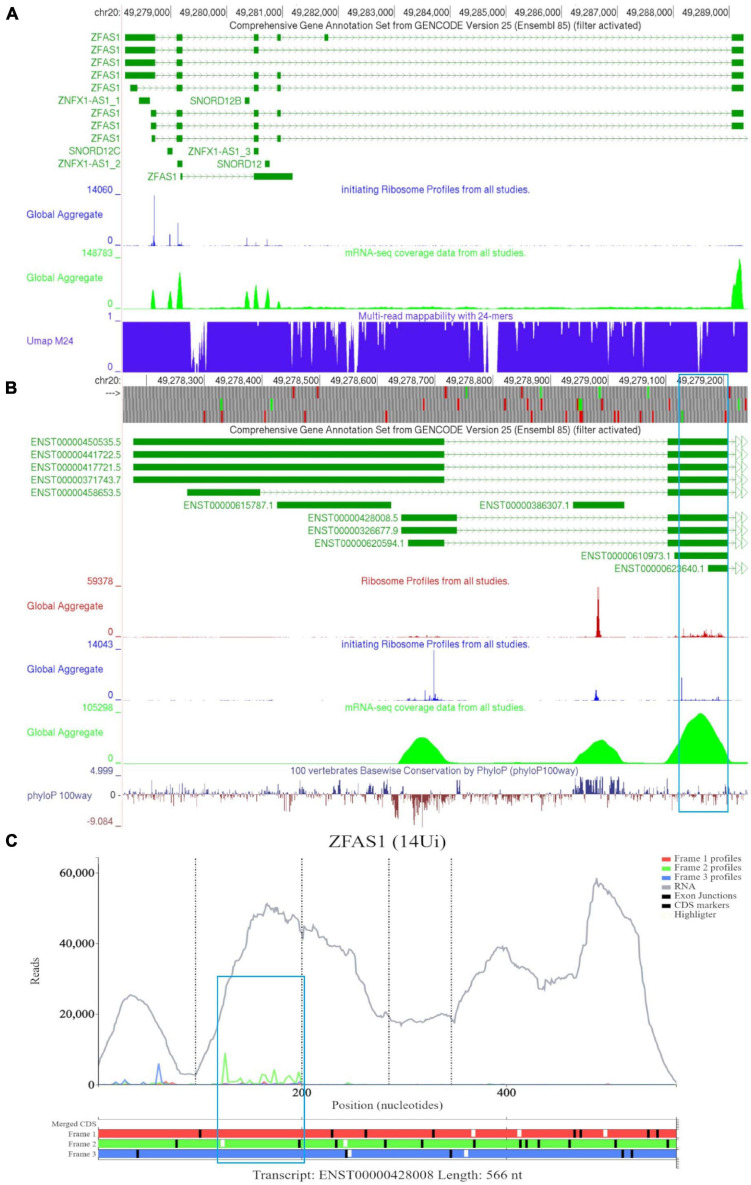
Visualization of *ZFAS1* in GWIPS-viz and Trips-Viz. **(A)** Whole transcript view of *ZFAS1* in GWIPS-viz with the ‘Comprehensive Annotation Set from Gencode Version 25,’ ‘initiating Ribosome Profiles from all studies,’ ‘mRNA-seq coverage data from all studies’ and ‘Multi-read mappability with 24mers’ tracks enabled. **(B)** Visualization of the 5′ end of *ZFAS1* in GWIPS-viz with the tracks described in Section Setup and Configurations enabled. An ORF on the second exon of multiple *ZFAS1* isoforms is outlined in blue. **(C)** Visualization of *ZFAS1* isoform ENST00000428008 in Trips-viz. The ORF previously outlined in blue in GWIPS-viz is similarly outlined again.

In Figure 6A, we observed that there is a lack of RNA-seq data corresponding to the 5′ end of the longer *ZFAS1* isoforms (ENST00000450535, ENST00000441722, ENST00000417721, and ENST00000371743). To explore whether this is potentially due to mapping artifacts, we enabled the track “Multi-read mappability with 24mers.” The Umap track represents the probability that a randomly selected read of k-length (24 base pairs is the default) that overlaps a given position in the unconverted genome is uniquely mappable (Karimzadeh et al., 2018). According to the track the mappability is high in this region. We also noted high footprint peaks of initiating ribosomes at the first two exons of the shorter isoforms (ENST00000428008 and ENST00000326677). The 5′ parts of these transcripts are visualized at a closer zoom in Figure 6B. The first high footprint peak of initiating ribosomes occurs on the first exon of the shorter isoforms but does not appear to match any ATG sites. The second peak of initiating ribosomes matches the sixth ATG which is located on *SNORD12C* (ENST00000386307). There also appears to be high peak of elongating ribosomes at this locus. As expected for a snoRNA *SNORD12C* sequence is highly conserved as can be seen in the PhyloP track.

**FIGURE 7:**
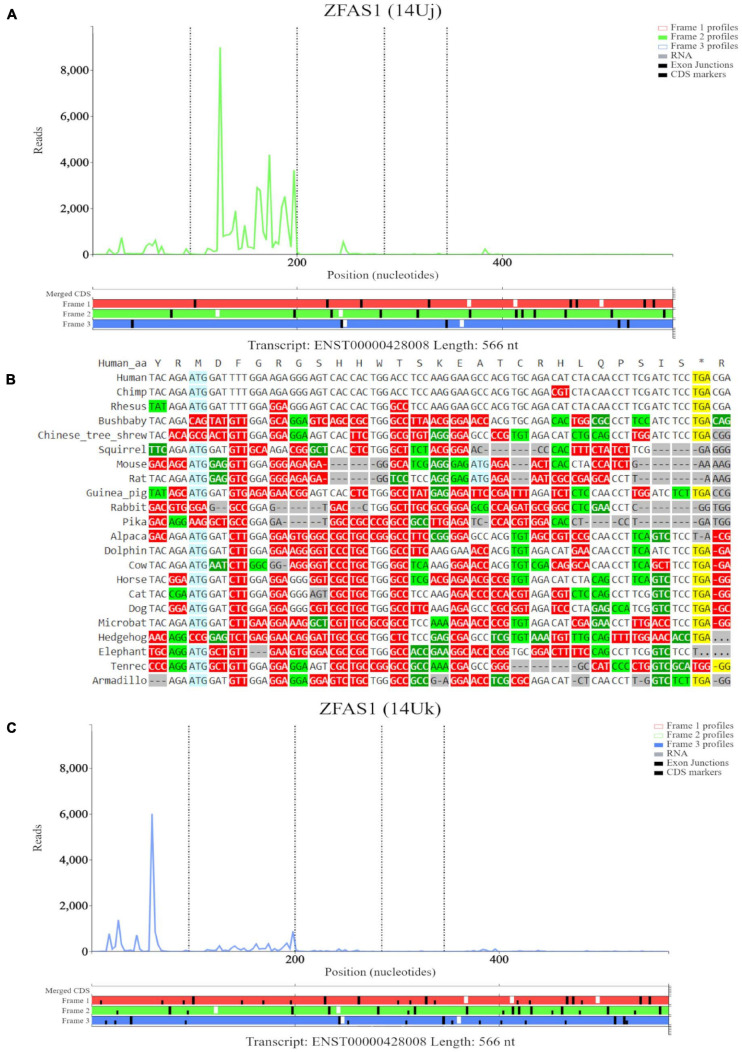
Visualization of *ZFAS1* in Trips-Viz and CodAlignView. **(A)** Visualization of transcript ENST00000428008 without RNA-seq data enabled and only reading frame 2 enabled. **(B)** CodAlignView for the ORF on *ZFAS1* showing a high ratio of radical non-synonymous codon substitutions. **(C)** Visualization of transcript ENST00000428008 without RNA-seq data enabled and only reading frame 3 enabled. Alternative start sequences of CTG and GTG were enabled and are represented by the black dashes.

The third high footprint peak of initiating ribosomes matches the eighth ATG site. Multiple transcript isoforms appear to contain this ORF, which is outlined in a blue rectangle (Figure 6B). Poor nucleotide conservation is seen for this sequence on the PhyloP track. RNAseq data support existence of ENST00000428008 and ENST00000326677 transcript isoforms. This ATG is the first ATG from the 5′ end in these transcripts. We elected to examine transcript ENST00000428008 in Trips-Viz (Figure 6C). There is a good support for translation of the ORF that starts with this ATG in the corresponding reading frame (green). The corresponding region is shown at a closer zoom in Figure 7A (RNA-seq data disabled and only reading frame two enabled). However, codon substitution patterns do not support selection typical for protein coding evolution for this ORF (Figure 7B).

Additionally, there were footprints upstream of the first ATG that were biased to reading frame three (blue) on Trips-viz that match the first high footprint peak of initiating ribosomes seen in GWIPS-viz (Figure 6B). However, when looking specifically only at footprints supporting frame three (Figure 7C, short black dashes show positions of near cognate start codons CTG and GTG). It is clear that these footprints are not contained within a single ORF and span the area containing a stop codon in this reading frame. Thus, these protected fragments are unlikely to derive from actively translated ribosomes. We further visualized sequencing reads aligned to *SNORD12C* using Trips-Viz (Supplementary Figure 3B). The distribution of sequencing reads does not exhibit good triplet periodicity and their positions do not match a particular ORF. Like with other snoRNAs, these fragments are unlikely to be genuine ribosomal footprints. It is more likely that they originate from ribonucleprotein or ribosomal complexes according to snoRNAs role in ribosomal RNA processing (Sloan et al., 2017).

Lastly, we examined X Inactive Specific Transcript (*XIST*), a nuclear lncRNA with over 19,000 nucleotides (19,296) in humans and located on the q arm of the X chromosome. Previous work proposed that *XIST* evolved in eutherians from the pseudogenization of a protein coding gene (Duret et al., 2006). Following this, another study suggested *XIST* had dual origins, namely pseudogenization of a protein coding gene and a set of transposable elements. Specifically, the *XIST* promoter region and four exons in eutherians retained homology to exons of the protein coding *LNX3* gene, while the other six exons were similar to different transposable elements (Elisaphenko et al., 2008). The authors further suggest that the *XIST* gene lost the coding functions of *LNX3* gene, but due to transposon insertions and subsequent partial amplification, formed new functional domains. These new domains are now believed to be necessary for its role in the silencing of X-chromosome genes (Elisaphenko et al., 2008; Romito and Rougeulle, 2011).

**FIGURE 8:**
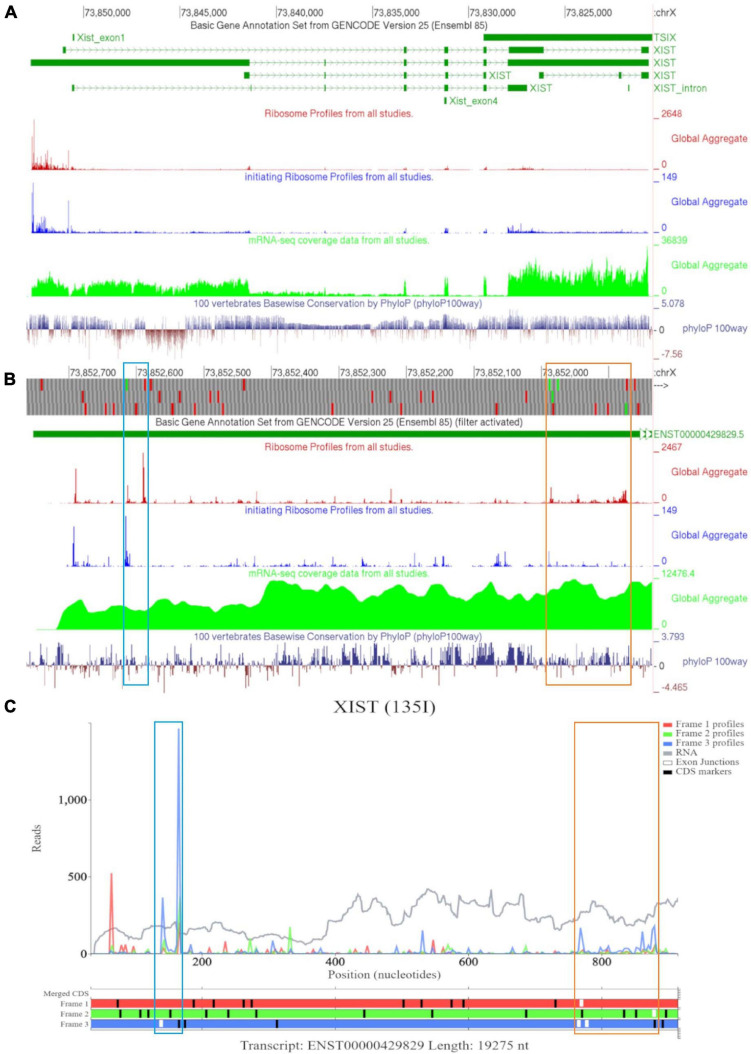
Visualization of *XIST* in GWIPS-viz and Trips-Viz. **(A)** Whole transcript view of *XIST* in GWIPS-viz with the tracks described in Section Setup and Configurations enabled. **(B)** Visualization of the 5′ end of *XIST* isoform ENST00000429829 in GWIPS-viz with the tracks described in Section Setup and Configurations enabled. Two translated ORFs are outlined in blue and orange, respectively. **(C)** Visualization of the 5′ end of transcript ENST00000429829 in Trips-Viz. The two translated ORFs previously outlined in blue and orange on GWIPS-viz are similarly outlined again.

Examining *XIST* on GWIPS-viz revealed a long transcript on the reverse strand (Figure 8A). Dense ribosomal peaks and footprints are noted at the 5′ end of the long isoform ENST00000429829. Available RNA-seq data further supports that the long isoform is transcribed. Zooming in to the area of dense footprints (Figure 8B), showed a high footprint peak of initiating ribosomes that matches the first ATG. The ORF at this locus, outlined in blue, is very short (30nt), the distribution of footprints is consistent with its translation The second ATG also matches a footprint peak of initiating ribosomes. The ORF at this locus is outlined in orange and shows elongating ribosomes mapped to it.

**FIGURE 9:**
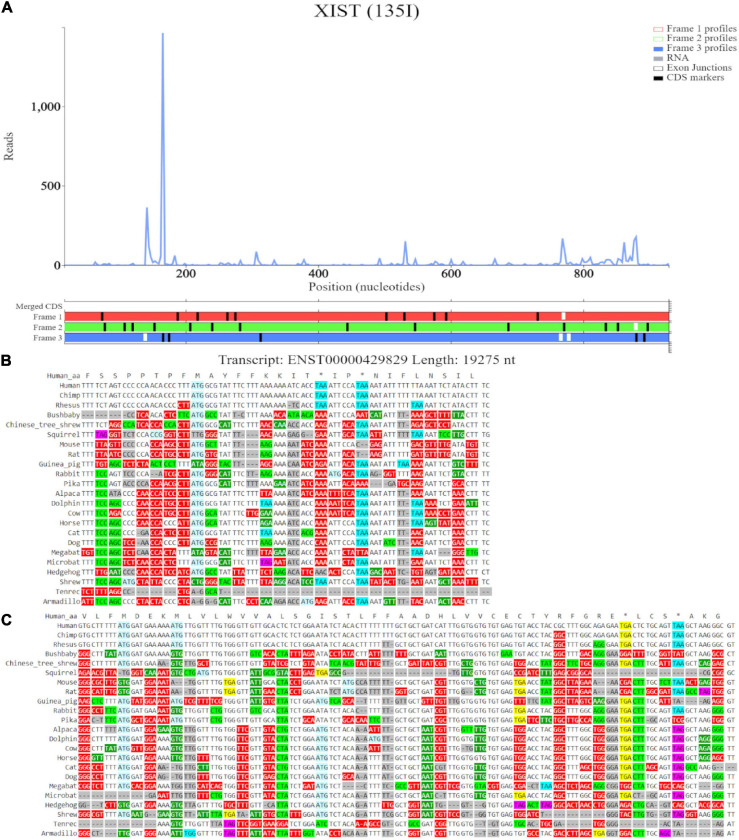
Visualization of *XIST* in Trips-Viz and CodAlignView. **(A)** Visualization of transcript ENST00000429829 without RNA-seq data enabled and only reading frame 3 enabled. **(B)** CodAlignView for the ORF previously outlined in blue showing predominantly absolute codon conservation. **(C)** CodAlignView for the ORF previously outlined in orange showing mostly absolute codon conservation, there are also more radical non-synonymous codon substitutions than synonymous substitutions.

Trips-Viz visualization of the data for this region of transcript ENST00000429829 is shown in Figure 8C. Translation of ORFs initiated at the first and second ATGs is supported with good triplet periodicity matching expected reading frame three (blue) in both cases. Figure 9A shows distribution of footprints that support only these reading frames. We could see an increase of footprint densities in these ORF that exceeds background. However, the codon substitution patterns do not support protein coding evolution for both ORFs (Figures 9B, C, respectively).

## Final Thoughts

Here we used examples of lncRNAs with reported translated ORFs to guide in the manual examination of publicly available ribosome profiling data using Trips-viz and GWIPS-viz. CodAlignView was then used for detailed examination of codon substitution patterns as evidence for evolutionary selection acting on potential protein coding sequences. We used *MTLN* as an example of genuine protein coding RNA and illustrated typical features of ribosome profiling data and codon substitution patterns associated with genuine ORF translation and protein coding evolution. Expression of lncRNAs is highly specific (Hon et al., 2017; Douka et al., 2021), therefore a long RNA isoform of *MTLN* (ENST00000414416) may be expressed in some cells, however, translation of such mRNA is unlikely to produce *MTLN* proteoforms since its start codon cannot be reached by scanning preinitiation complex.

RNA component of RNase P encoded by *RPPH1* was used as a negative example to demonstrate the patterns that are inconsistent with translation and protein evolution. Finally, we examined the data available for other lncRNAs with reported translated ORFs, i.e., *SNHG8* and *ZFAS1* and *XIST* and concluded that they contain multiple short ORFs that are likely translated even though they do not exhibit signatures of protein coding evolution. We can only speculate on the biological significance of translation of these short ORFs. We do not know if they code any stable and biologically active peptides, as there is no support for their evolutionary selection. Yet it is possible that they could be used by the immune system as antigens for self-recognition. Additionally the translation of these ORFs may influence processing, stability, localization and structural folding of the corresponding lncRNAs irrespective of biological significance of the products of this translation.

Because of the complexity of translation and of ribosome profiling data, it is very difficult to design automatic tools for translation detections that are highly accurate. Thus, we hope that manual examination of individual cases using the tools described here, will benefit researchers in examining translation status of individual ORFs in non-coding RNAs.

## Data Availability Statement

Publicly available datasets were analyzed in this study. This data can be found in Trips-viz which is freely available at https://trips.ucc.ie. Source code for Trips-viz is available at https://github.com/skiniry/Trips-viz. Gwips-viz is freely available at https://gwips.ucc.ie/. CodAlignView is freely available at https://data.broadinstitute.org/compbio1/cav.php.

## Author Contributions

OZ: data curation, visualization, and writing. SK: software, data curation, and writing. PB: conceptualization, supervision, writing, project administration, and funding acquisition. KD: supervision, writing, project administration, and funding acquisition. All authors contributed to the article and approved the submitted version.

## Conflict of Interest

PB is a founder of Ribomaps Ltd., a company that provides ribosome profiling as a service. The remaining authors declare that the research was conducted in the absence of any commercial or financial relationships that could be construed as a potential conflict of interest.

## Publisher’s Note

All claims expressed in this article are solely those of the authors and do not necessarily represent those of their affiliated organizations, or those of the publisher, the editors and the reviewers. Any product that may be evaluated in this article, or claim that may be made by its manufacturer, is not guaranteed or endorsed by the publisher.

## Footnotes

1
https://trips.ucc.ie

2
https://data.broadinstitute.org/compbio1/cav.php

## References

[B1] Anderson D. M. Anderson K. M. Chang C. L. Makarewich C. A. Nelson B. R. McAnally J. R. (2015). A micropeptide encoded by a putative long noncoding RNA regulates muscle performance. *Cell* 160 595–606. 10.1016/j.cell.2015.01.009 25640239 PMC4356254

[B2] Andreev D. E. O’Connor P. B. F. Loughran G. Dmitriev S. E. Baranov P. V. Shatsky I. N. (2017). Insights into the mechanisms of eukaryotic translation gained with ribosome profiling. *Nucleic Acids Res.* 45 513–526. 10.1093/nar/gkw1190 27923997 PMC5314775

[B3] Askarian-Amiri M. E. Crawford J. French J. D. Smart C. E. Smith M. A. Clark M. B. (2011). SNORD-host RNA Zfas1 is a regulator of mammary development and a potential marker for breast cancer. *RNA* 17 878–891. 10.1261/rna.2528811 21460236 PMC3078737

[B4] Baranov P. V. Michel A. M. (2016). Illuminating translation with ribosome profiling spectra. *Nat. Methods* 13 123–124. 10.1038/nmeth.3738 26820545

[B5] Benitez-Cantos M. S. Yordanova M. M. O’Connor P. B. F. Zhdanov A. V. Kovalchuk S. I. Papkovsky D. B. (2020). Translation initiation downstream from annotated start codons in human mRNAs coevolves with the Kozak context. *Genome Res.* 30 974–984. 10.1101/gr.257352.119 32669370 PMC7397870

[B6] Birney E. Stamatoyannopoulos J. A. Dutta A. Guigó R. Gingeras T. R. Margulies E. H. (2007). Identification and analysis of functional elements in 1% of the human genome by the ENCODE pilot project. *Nature* 447 799–816. 10.1038/nature05874 17571346 PMC2212820

[B7] Brar G. A. Weissman J. S. (2015). Ribosome profiling reveals the what, when, where and how of protein synthesis. *Nat. Rev. Mol. Cell Biol.* 16 651–664. 10.1038/nrm4069 26465719 PMC5522010

[B8] Brunet M. A. Brunelle M. Lucier J. F. Delcourt V. Levesque M. Grenier F. (2019). OpenProt: a more comprehensive guide to explore eukaryotic coding potential and proteomes. *Nucleic Acids Res.* 47 D403–D410. 10.1093/nar/gky936 30299502 PMC6323990

[B9] Calviello L. Mukherjee N. Wyler E. Zauber H. Hirsekorn A. Selbach M. (2016). Detecting actively translated open reading frames in ribosome profiling data. *Nat. Methods* 13 165–170. 10.1038/nmeth.3688 26657557

[B10] Catherman A. D. Li M. Tran J. C. Durbin K. R. Compton P. D. Early B. P. (2013). Top down proteomics of human membrane proteins from enriched mitochondrial fractions. *Anal. Chem.* 85 1880–1888. 10.1021/ac3031527 23305238 PMC3565750

[B11] Chen J. Brunner A. D. Cogan J. Z. Nuñez J. K. Fields A. P. Adamson B. (2020). Pervasive functional translation of noncanonical human open reading frames. *Science* 367 140–146. 10.1126/science.aav5912 32139545 PMC7289059

[B12] Cheng J. Kapranov P. Drenkow J. Dike S. Brubaker S. Patel S. (2005). Transcriptional maps of 10 human chromosomes at 5-nucleotide resolution. *Science* 308 1149–1154. 10.1126/science.1108625 15790807

[B13] Chew G. L. Pauli A. Rinn J. L. Regev A. Schier A. F. Valen E. (2013). Ribosome profiling reveals resemblance between long non-coding RNAs and 5′ leaders of coding RNAs. *Development* 140 2828–2834. 10.1242/dev.098343 23698349 PMC3678345

[B14] Chugunova A. Loseva E. Mazin P. Mitina A. Navalayeu T. Bilan D. (2019). LINC00116 codes for a mitochondrial peptide linking respiration and lipid metabolism. *Proc. Natl. Acad. Sci. U.S.A.* 116 4940–4945. 10.1073/pnas.1809105116 30796188 PMC6421467

[B15] Crappé J. Ndah E. Koch A. Steyaert S. Gawron D. De Keulenaer S. (2015). PROTEOFORMER: deep proteome coverage through ribosome profiling and MS integration. *Nucleic Acids Res.* 43:e29. 10.1093/nar/gku1283 25510491 PMC4357689

[B16] da Veiga Leprevost F. Haynes S. E. Avtonomov D. M. Chang H. Y. Shanmugam A. K. Mellacheruvu D. (2020). Philosopher: a versatile toolkit for shotgun proteomics data analysis. *Nat. Methods* 17 869–870. 10.1038/s41592-020-0912-y 32669682 PMC7509848

[B17] D’Lima N. G. Ma J. Winkler L. Chu Q. Loh K. H. Corpuz E. O. (2017). A human microprotein that interacts with the mRNA decapping complex. *Nat. Chem. Biol.* 13 174–180. 10.1038/nchembio.2249 27918561 PMC5247292

[B18] Douka K. Birds I. Wang D. Kosteletos A. Clayton S. Byford A. (2021). Cytoplasmic long non-coding RNAs are differentially regulated and translated during human neuronal differentiation. *RNA* [Epub ahead of print]. 10.1261/rna.078782.121 34193551 PMC8370745

[B19] Duret L. Chureau C. Samain S. Weissanbach J. Avner P. (2006). The Xist RNA gene evolved in eutherians by pseudogenization of a protein-coding gene. *Science* 312 1653–1655. 10.1126/science.1126316 16778056

[B20] Elisaphenko E. A. Kolesnikov N. N. Shevchenko A. I. Rogozin I. B. Nesterova T. B. Brockdorff N. (2008). A dual origin of the Xist gene from a protein-coding gene and a set of transposable elements. *PLoS One* 3:e2521. 10.1371/journal.pone.0002521 18575625 PMC2430539

[B21] Erhard F. Halenius A. Zimmermann C. L’Hernault A. Kowalewski D. J. Weekes M. P. (2018). Improved Ribo-seq enables identification of cryptic translation events. *Nat. Methods* 15 363–366. 10.1038/nmeth.4631 29529017 PMC6152898

[B22] Evans D. Marquez S. M. Pace N. R. (2006). RNase P: interface of the RNA and protein worlds. *Trends Biochem. Sci.* 31, 333–341. 10.1016/j.tibs.2006.04.007 16679018

[B23] Fang Y. Fullwood M. J. (2016). Roles, functions, and mechanisms of long non-coding RNAs in cancer. *Genomics Proteomics Bioinform.* 14 42–54. 10.1016/j.gpb.2015.09.006 26883671 PMC4792843

[B24] Fields A. P. Rodriguez E. H. Jovanovic M. Stern-Ginossar N. Haas B. J. Mertins P. (2015). A regression-based analysis of ribosome-profiling data reveals a conserved complexity to mammalian translation. *Mol. Cell* 60 816–827. 10.1016/j.molcel.2015.11.013 26638175 PMC4720255

[B25] Fija-Lkowska D. Verbruggen S. Ndah E. Jonckheere V. Menschaert G. Van Damme P. (2017). EIF1 modulates the recognition of suboptimal translation initiation sites and steers gene expression via uORFs. *Nucleic Acids Res.* 45 7997–8013. 10.1093/nar/gkx469 28541577 PMC5570006

[B26] Friesen M. Warren C. R. Yu H. Toyohara T. Ding Q. Florido M. H. C. (2020). Mitoregulin controls β-oxidation in human and mouse adipocytes. *Stem Cell Rep.* 14 590–602. 10.1016/j.stemcr.2020.03.002 32243843 PMC7160386

[B27] Gaertner B. van Heesch S. Schneider-Lunitz V. Schulz J. F. Witte F. Blachut S. (2020). A human esc-based screen identifies a role for the translated lncrna linc00261 in pancreatic endocrine differentiation. *eLife* 9:58659. 10.7554/ELIFE.58659 32744504 PMC7423336

[B28] Gameiro P. A. Struhl K. (2018). Nutrient deprivation elicits a transcriptional and translational inflammatory response coupled to decreased protein synthesis. *Cell Rep.* 24 1415–1424. 10.1016/j.celrep.2018.07.021 30089253 PMC6419098

[B29] Goodarzi H. Nguyen H. C. B. Zhang S. Dill B. D. Molina H. Tavazoie S. F. (2016). Modulated expression of specific tRNAs drives gene expression and cancer progression. *Cell* 165 1416–1427. 10.1016/j.cell.2016.05.046 27259150 PMC4915377

[B30] Großhans H. Filipowicz W. (2008). Molecular biology: the expanding world of small RNAs. *Nature* 451 414–416. 10.1038/451414a 18216846

[B31] Guo B. Wu S. Zhu X. Zhang L. Deng J. Li F. (2020). Micropeptide CIP 2A- BP encoded by LINC 00665 inhibits triple-negative breast cancer progression. *EMBO J.* 39:e102190. 10.15252/embj.2019102190 31755573 PMC6939193

[B32] Guo J. U. Agarwal V. Guo H. Bartel D. P. (2014). Expanded identification and characterization of mammalian circular RNAs. *Genome Biol.* 15:409. 10.1186/s13059-014-0409-z 25070500 PMC4165365

[B33] Guttman M. Russell P. Ingolia N. T. Weissman J. S. Lander E. S. (2013). Ribosome profiling provides evidence that large noncoding RNAs do not encode proteins. *Cell* 154 240–251. 10.1016/j.cell.2013.06.009 23810193 PMC3756563

[B34] Hon C. C. Ramilowski J. A. Harshbarger J. Bertin N. Rackham O. J. L. Gough J. (2017). An atlas of human long non-coding RNAs with accurate 5′ ends. *Nature* 543 199–204. 10.1038/nature21374 28241135 PMC6857182

[B35] Ingolia N. T. (2014). Ribosome profiling: new views of translation, from single codons to genome scale. *Nat. Rev. Genet.* 15 205–213. 10.1038/nrg3645 24468696

[B36] Ingolia N. T. Brar G. A. Stern-Ginossar N. Harris M. S. Talhouarne G. J. S. Jackson S. E. (2014). Ribosome profiling reveals pervasive translation outside of annotated protein-coding genes. *Cell Rep.* 8 1365–1379. 10.1016/j.celrep.2014.07.045 25159147 PMC4216110

[B37] Ingolia N. T. Ghaemmaghami S. Newman J. R. S. Weissman J. S. (2009). Genome-wide analysis in vivo of translation with nucleotide resolution using ribosome profiling. *Science* 324 218–223. 10.1126/science.1168978 19213877 PMC2746483

[B38] Ingolia N. T. Hussmann J. A. Weissman J. S. (2019). Ribosome profiling: global views of translation. *Cold Spring Harb. Perspect. Biol.* 11:a032698. 10.1101/cshperspect.a032698 30037969 PMC6496350

[B39] Ingolia N. T. Lareau L. F. Weissman J. S. (2011). Ribosome profiling of mouse embryonic stem cells reveals the complexity and dynamics of mammalian proteomes. *Cell* 147 789–802. 10.1016/j.cell.2011.10.002 22056041 PMC3225288

[B40] Iwasaki S. Floor S. N. Ingolia N. T. (2016). Rocaglates convert DEAD-box protein eIF4A into a sequence-selective translational repressor. *Nature* 534 558–561. 10.1038/nature17978 27309803 PMC4946961

[B41] Ji Z. (2018). RibORF: identifying genome-wide translated open reading frames using ribosome profiling. *Curr. Protoc. Mol. Biol.* 124:67. 10.1002/cpmb.67 30178897 PMC6168376

[B42] Ji Z. Song R. Huang H. Regev A. Struhl K. (2016). Transcriptome-scale RNase-footprinting of RNA-protein complexes. *Nat. Biotechnol.* 34 410–413. 10.1038/nbt.3441 26900662 PMC4824641

[B43] Ji Z. Song R. Regev A. Struhl K. (2015). Many lncRNAs, 5′UTRs, and pseudogenes are translated and some are likely to express functional proteins. *eLife* 4:e08890. 10.7554/eLife.08890 26687005 PMC4739776

[B44] Jungreis I. Lin M. Kellis M. (2021). *CodAlignView: A Tool For Visualizing Protein-Coding Constraint.* Available online at: https://data.broadinstitute.org/compbio1/CodAlignViewUsersGuide.html (accessed January 14, 2021).

[B45] Karimzadeh M. Ernst C. Kundaje A. Hoffman M. M. (2018). Umap and Bismap: quantifying genome and methylome mappability. *Nucleic Acids Res.* 46:e120. 10.1093/nar/gky677 30169659 PMC6237805

[B46] Kiniry S. J. Judge C. E. Michel A. M. Baranov P. V. (2021). Trips-Viz: an environment for the analysis of public and user-generated ribosome profiling data. *Nucleic Acids Res.* 49 W662–W670. 10.1093/nar/gkab323 33950201 PMC8262740

[B47] Kiniry S. J. Michel A. M. Baranov P. V. (2020). Computational methods for ribosome profiling data analysis. *Wiley Interdiscip. Rev. RNA* 11:1577. 10.1002/wrna.1577 31760685

[B48] Kiniry S. J. O’Connor P. B. F. Michel A. M. Baranov P. V. (2019). Trips-Viz: a transcriptome browser for exploring Ribo-Seq data. *Nucleic Acids Res.* 47 D847–D852. 10.1093/nar/gky842 30239879 PMC6324076

[B49] Kong A. T. Leprevost F. V. Avtonomov D. M. Mellacheruvu D. Nesvizhskii A. I. (2017). MSFragger: ultrafast and comprehensive peptide identification in mass spectrometry-based proteomics. *Nat. Methods* 14 513–520. 10.1038/nmeth.4256 28394336 PMC5409104

[B50] Lee S. Liu B. Lee S. Huang S. X. Shen B. Qian S. B. (2012). Global mapping of translation initiation sites in mammalian cells at single-nucleotide resolution. *Proc. Natl. Acad. Sci. U.S.A.* 109 E2424–E2432. 10.1073/pnas.1207846109 22927429 PMC3443142

[B51] Lin M. F. Jungreis I. Kellis M. (2011). PhyloCSF: a comparative genomics method to distinguish protein coding and non-coding regions. *Bioinformatics* 27 i275–i282. 10.1093/bioinformatics/btr209 21685081 PMC3117341

[B52] Lin Y. F. Xiao M. H. Chen H. X. Meng Y. Zhao N. Yang L. (2019). A novel mitochondrial micropeptide MPM enhances mitochondrial respiratory activity and promotes myogenic differentiation. *Cell Death Dis.* 10 1–11. 10.1038/s41419-019-1767-y 31296841 PMC6624212

[B53] Loughran G. Chou M. Y. Ivanov I. P. Jungreis I. Kellis M. Kiran A. M. (2014). Evidence of efficient stop codon readthrough in four mammalian genes. *Nucleic Acids Res.* 42 8928–8938. 10.1093/nar/gku608 25013167 PMC4132726

[B54] Martinez T. F. Chu Q. Donaldson C. Tan D. Shokhirev M. N. Saghatelian A. (2020). Accurate annotation of human protein-coding small open reading frames. *Nat. Chem. Biol.* 16 458–468. 10.1038/s41589-019-0425-0 31819274 PMC7085969

[B55] Michel A. M. Andreev D. E. Baranov P. V. (2014a). Computational approach for calculating the probability of eukaryotic translation initiation from ribo-seq data that takes into account leaky scanning. *BMC Bioinformatics* 15:380. 10.1186/s12859-014-0380-4 25413677 PMC4245810

[B56] Michel A. M. Choudhury K. R. Firth A. E. Ingolia N. T. Atkins J. F. Baranov P. V. (2012). Observation of dually decoded regions of the human genome using ribosome profiling data. *Genome Res.* 22 2219–2229. 10.1101/gr.133249.111 22593554 PMC3483551

[B57] Michel A. M. Fox G. Kiran M. De Bo C. O’Connor P. B. F. Heaphy S. M. (2014b). GWIPS-viz: development of a ribo-seq genome browser. *Nucleic Acids Res.* 42 D859–D864. 10.1093/nar/gkt1035 24185699 PMC3965066

[B58] Munzarová V. Pánek J. Gunišová S. Dányi I. Szamecz B. Valášek L. S. (2011). Translation reinitiation relies on the interaction between eIFa/TIF32 and progressively folded cis-acting mRNA elements preceding short uORFS. *PLoS Genet.* 7:e1002137. 10.1371/journal.pgen.1002137 21750682 PMC3131280

[B59] Navarro Gonzalez J. Zweig A. S. Speir M. L. Schmelter D. Rosenbloom K. R. Raney B. J. (2021). The UCSC genome browser database: 2021 update. *Nucleic Acids Res.* 49 D1046–D1057. 10.1093/nar/gkaa1070 33221922 PMC7779060

[B60] O’Connor P. B. F. Andreev D. E. Baranov P. V. (2016). Comparative survey of the relative impact of mRNA features on local ribosome profiling read density. *Nat. Commun.* 7:12915. 10.1038/ncomms12915 27698342 PMC5059445

[B61] Pang Y. Liu Z. Han H. Wang B. Li W. Mao C. (2020). Peptide SMIM30 promotes HCC development by inducing SRC/YES1 membrane anchoring and MAPK pathway activation. *J. Hepatol.* 73 1155–1169. 10.1016/j.jhep.2020.05.028 32461121

[B62] Park J. E. Yi H. Kim Y. Chang H. Kim V. N. (2016). Regulation of Poly(A) tail and translation during the somatic cell cycle. *Mol. Cell* 62 462–471. 10.1016/j.molcel.2016.04.007 27153541

[B63] Pollard K. S. Hubisz M. J. Rosenbloom K. R. Siepel A. (2010). Detection of nonneutral substitution rates on mammalian phylogenies. *Genome Res.* 20 110–121. 10.1101/gr.097857.109 19858363 PMC2798823

[B64] Raj A. Wang S. H. Shim H. Harpak A. Li Y. I. Engelmann B. (2016). Thousands of novel translated open reading frames in humans inferred by ribosome footprint profiling. *eLife* 5:e13328. 10.7554/eLife.13328 27232982 PMC4940163

[B65] Reuter K. Biehl A. Koch L. Helms V. (2016). PreTIS: a tool to predict non-canonical 5′ UTR translational initiation sites in human and mouse. *PLoS Comput. Biol.* 12:e1005170. 10.1371/journal.pcbi.1005170 27768687 PMC5074520

[B66] Romito A. Rougeulle C. (2011). Origin and evolution of the long non-coding genes in the X-inactivation center. *Biochimie* 93 1935–1942. 10.1016/j.biochi.2011.07.009 21820484

[B67] Rosenbloom K. R. Armstrong J. Barber G. P. Casper J. Clawson H. Diekhans M. (2015). The UCSC Genome Browser database: 2015 update. *Nucleic Acids Res.* 43 D670–D681. 10.1093/nar/gku1177 25428374 PMC4383971

[B68] Ruiz-Orera J. Albà M. M. (2019). Conserved regions in long non-coding RNAs contain abundant translation and protein–RNA interaction signatures. *NAR Gen. Bioinforma* 1:e2. 10.1093/nargab/lqz002 33575549 PMC7671363

[B69] Schramm L. Hernandez N. (2002). Recruitment of RNA polymerase III to its target promoters. *Genes Dev.* 16 2593–2620. 10.1101/gad.1018902 12381659

[B70] Shahrouki P. Larsson E. (2012). The non-coding oncogene: a case of missing DNA evidence? *Front. Genet.* 3:170. 10.3389/fgene.2012.00170 22988449 PMC3439828

[B71] Sloan K. E. Warda A. S. Sharma S. Entian K. D. Lafontaine D. L. J. Bohnsack M. T. (2017). Tuning the ribosome: the influence of rRNA modification on eukaryotic ribosome biogenesis and function. *RNA Biol.* 14 1138–1152. 10.1080/15476286.2016.1259781 27911188 PMC5699541

[B72] Smith C. M. Steitz J. A. (1998). Classification of gas5 as a multi-small-nucleolar-RNA (snoRNA) host gene and a member of the 5′-terminal oligopyrimidine gene family reveals common features of snoRNA host genes. *Mol. Cell. Biol.* 18 6897–6909. 10.1128/mcb.18.12.6897 9819378 PMC109273

[B73] Stein C. S. Jadiya P. Zhang X. McLendon J. M. Abouassaly G. M. Witmer N. H. (2018). Mitoregulin: a lncRNA-encoded microprotein that supports mitochondrial supercomplexes and respiratory efficiency. *Cell Rep.* 23 3710.e8–3720.e8. 10.1016/j.celrep.2018.06.002 29949756 PMC6091870

[B74] Storz G. (2002). An expanding universe of noncoding RNAs. *Science* 296 1260–1263. 10.1126/science.1072249 12016301

[B75] Sun Y. H. Zhu J. Xie L. H. Li Z. Meduri R. Zhu X. (2020). Ribosomes guide pachytene piRNA formation on long intergenic piRNA precursors. *Nat. Cell Biol.* 22 200–212. 10.1038/s41556-019-0457-4 32015435 PMC8041231

[B76] Tycowski K. T. Di Shu M. Steitz J. A. (1996). A mammalian gene with introns instead of exons generating stable RNA products. *Nature* 379 464–466. 10.1038/379464a0 8559254

[B77] van Bakel H. Nislow C. Blencowe B. J. Hughes T. R. (2010). Most “dark matter” transcripts are associated with known genes. *PLoS Biol.* 8:e1000371. 10.1371/journal.pbio.1000371 20502517 PMC2872640

[B78] van Heesch S. Witte F. Schneider-Lunitz V. Schulz J. F. Adami E. Faber A. B. (2019). The translational landscape of the human heart. *Cell* 178 242.e29–260.e29. 10.1016/j.cell.2019.05.010 31155234

[B79] Washietl S. Pedersen J. S. Korbel J. O. Stocsits C. Gruber A. R. Hackermüller J. (2007). Structured RNAs in the ENCODE selected regions of the human genome. *Genome Res.* 17 852–864. 10.1101/gr.5650707 17568003 PMC1891344

[B80] Weinberg D. E. Shah P. Eichhorn S. W. Hussmann J. A. Plotkin J. B. Bartel D. P. (2016). Improved ribosome-footprint and mRNA measurements provide insights into dynamics and regulation of yeast translation. *Cell Rep.* 14 1787–1799. 10.1016/j.celrep.2016.01.043 26876183 PMC4767672

[B81] Werner A. Iwasaki S. McGourty C. A. Medina-Ruiz S. Teerikorpi N. Fedrigo I. (2015). Cell-fate determination by ubiquitin-dependent regulation of translation. *Nature* 525 523–527. 10.1038/nature14978 26399832 PMC4602398

[B82] Wolfe A. L. Singh K. Zhong Y. Drewe P. Rajasekhar V. K. Sanghvi V. R. (2014). RNA G-quadruplexes cause eIF4A-dependent oncogene translation in cancer. *Nature* 513 65–70. 10.1038/nature13485 25079319 PMC4492470

[B83] Xiao Z. Huang R. Xing X. Chen Y. Deng H. Yang X. (2018). De novo annotation and characterization of the translatome with ribosome profiling data. *Nucleic Acids Res.* 46:e61. 10.1093/nar/gky179 29538776 PMC6007384

[B84] Xu B. Gogol M. Gaudenz K. Gerton J. L. (2016). Improved transcription and translation with L-leucine stimulation of mTORC1 in Roberts syndrome. *BMC Genomics* 17:25. 10.1186/s12864-015-2354-y 26729373 PMC4700579

[B85] Zhang P. He D. Xu Y. Hou J. Pan B. F. Wang Y. (2017). Genome-wide identification and differential analysis of translational initiation. *Nat. Commun.* 8 1–14. 10.1038/s41467-017-01981-8 29170441 PMC5701008

