# Supplementary figures and images for: Exploring Evidence of Non-coding RNA Translation With Trips-Viz and GWIPS-Viz Browsers
Source: Front Cell Dev Biol. 2021 Aug 12;9:703374. doi: 10.3389/fcell.2021.703374 (PMC8416628; doi:10.3389/fcell.2021.703374)

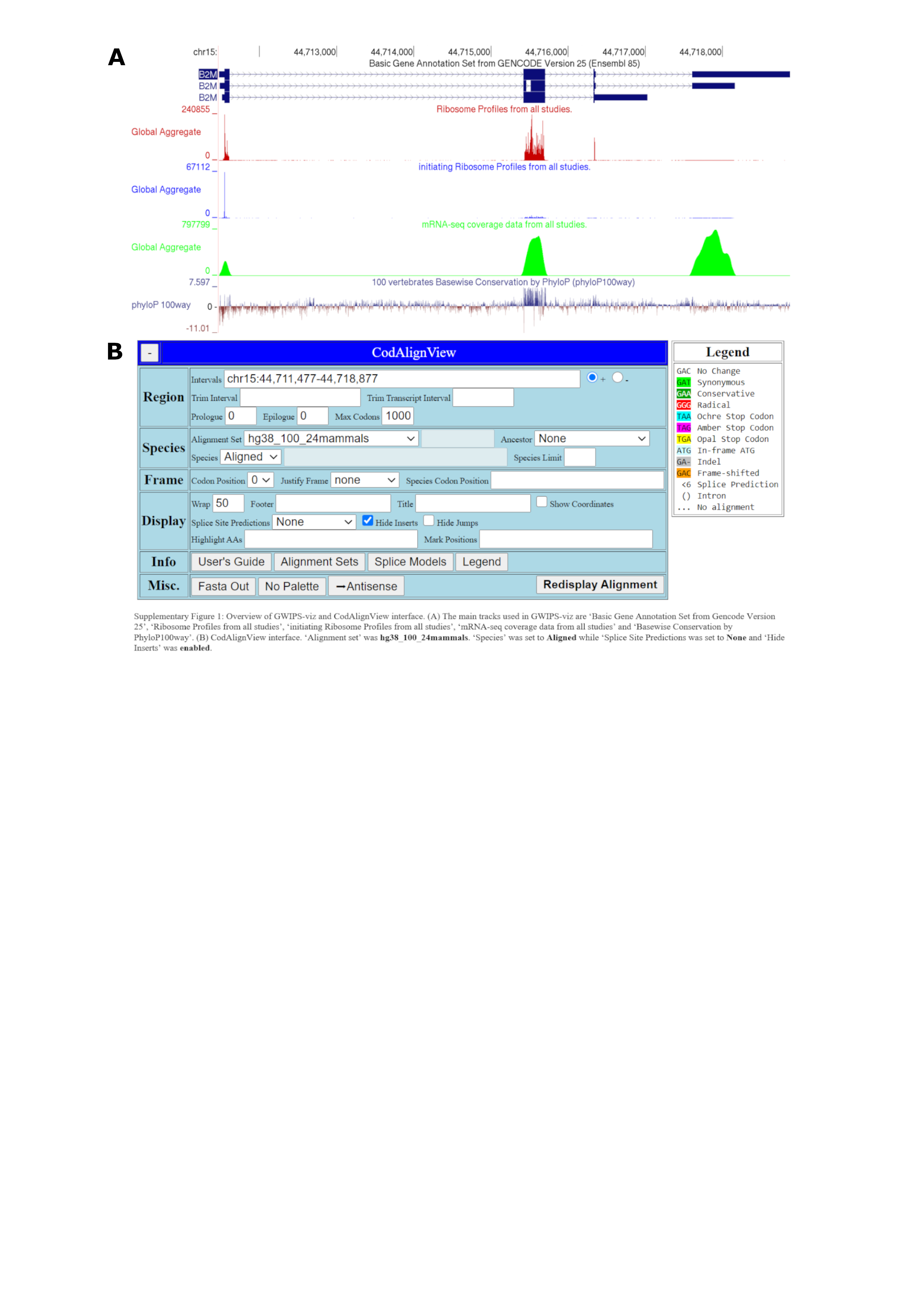

Supplement: Supplementary file 1 [file Image_1.JPEG]

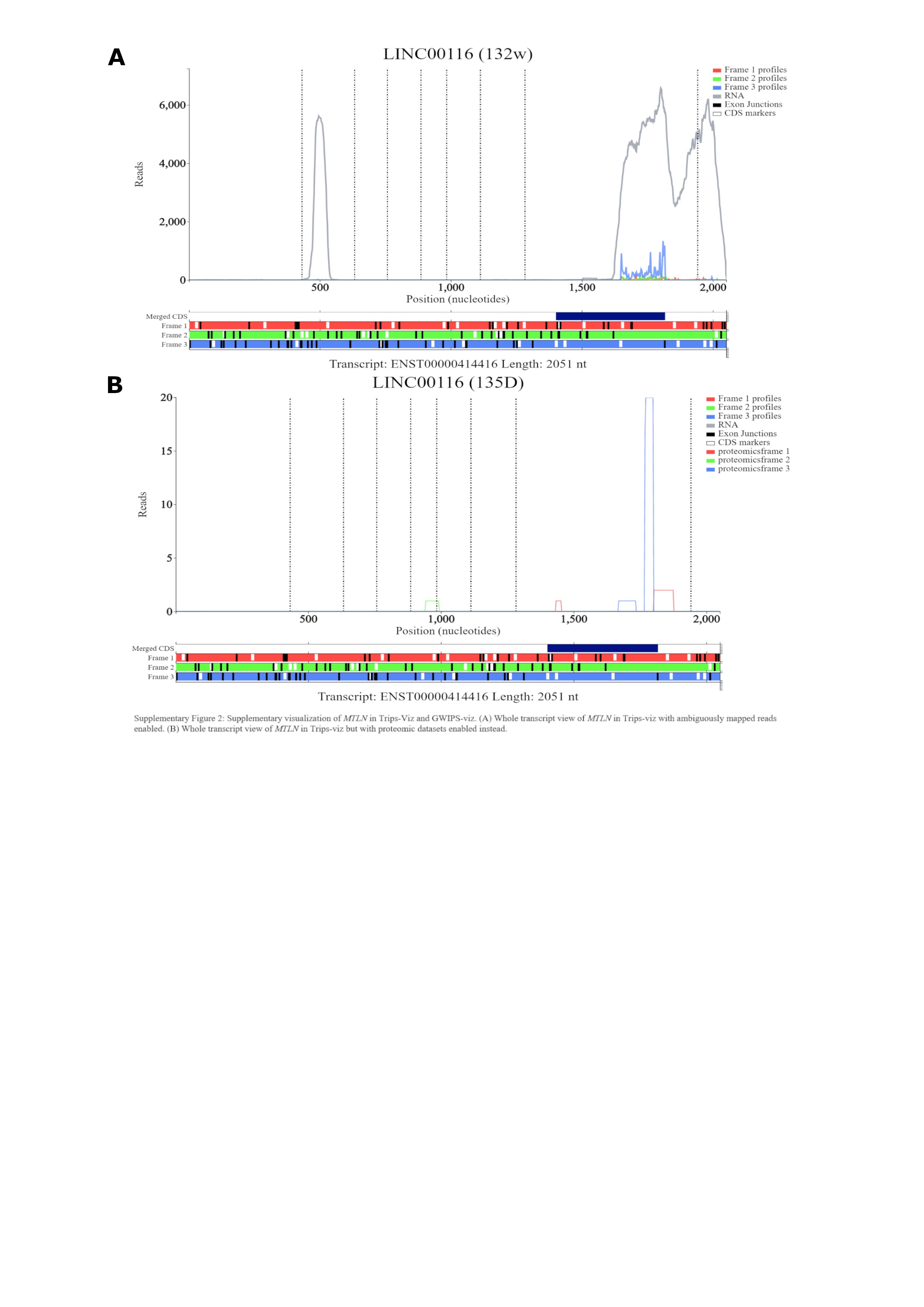

Supplement: Supplementary file 2 [file Image_2.JPEG]

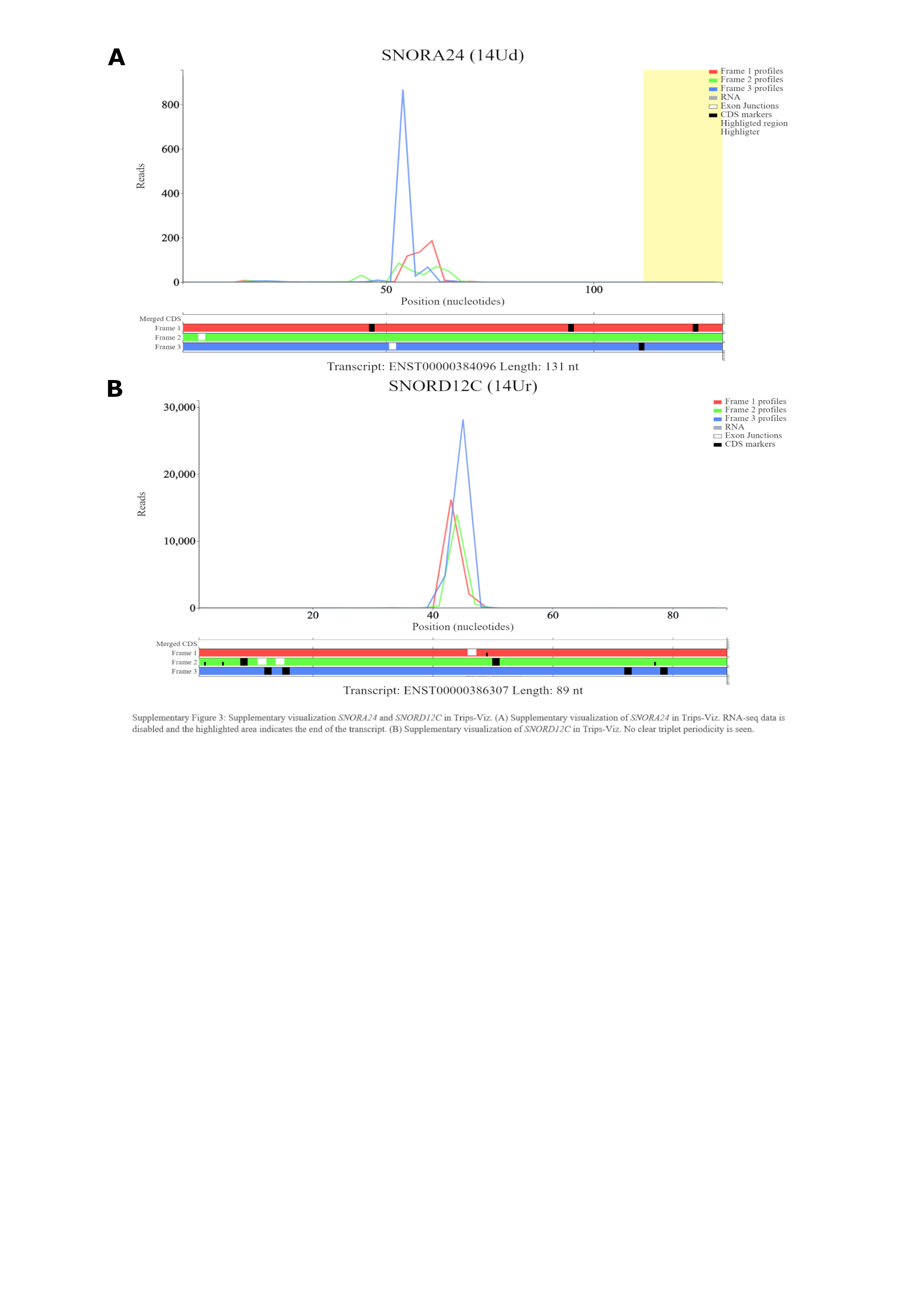

Supplement: Supplementary file 3 [file Image_3.JPEG]
